# Serum glial cell line-derived neurotrophic factor: a potential biomarker for white matter alteration in Parkinson’s disease with mild cognitive impairment

**DOI:** 10.3389/fnins.2024.1370787

**Published:** 2024-10-24

**Authors:** Yi Liu, Yan Xu, SuYan Tong

**Affiliations:** ^1^Department of Cell Biology and Neurobiology, Xuzhou Key Laboratory of Neurobiology, Xuzhou Medical University, Xuzhou, China; ^2^Xuzhou Children’s Hospital, Xuzhou, China; ^3^Department of Neurology, The Second Affiliated Hospital of Xuzhou Medical University, General Hospital of Xuzhou Mining Group, Xuzhou, Jiangsu, China

**Keywords:** Parkinson’s disease, mild cognitive impairment, glial cell line-derived neurotrophic factor, neuropsychological assessment, white matter

## Abstract

**Objective:**

Mild cognitive impairment (MCI) is a common non-motor manifestation of Parkinson’s disease, commonly referred to as PD-MCI. However, there is a lack of comprehensive data regarding the role of glial cell line-derived neurotrophic factor (GDNF) and cerebral white matter damage in the pathogenesis of PD-MCI. The objective of this study is to investigate the association between alterations in GDNF levels and cerebral white matter damage in individuals diagnosed with PD-MCI, as well as to explore their potential involvement in cognitive progression.

**Methods:**

Neuropsychological assessments were conducted on 105 patients with Parkinson’s disease and 45 healthy volunteers to examine various cognitive domains. An enzyme-linked immunosorbent assay (ELISA) was employed to measure serum levels of GDNF. Additionally, all participants underwent 3.0T magnetic resonance imaging (MRI) to acquire diffusion tensor images (DTI), and a voxel-based analysis (VBA) approach was utilized to compare the fractional anisotropy (FA) values of white matter in the brain.

**Results:**

There was a significant correlation between the right corpus callosum, right cingulate gyrus, and the Digit Span Backward Test (DSB-T) as well as the Trail Making Test A (TMT-A), both of which assess attention and working memory functions. The left internal capsule exhibited a significant correlation with the Trail Making Test B (TMT-B) and the Clock Drawing Test (CDT), which evaluate executive function. Additionally, the right cingulate gyrus showed a significant association with scores on the Auditory Verbal Learning Test-HuaShan (AVLT-H), assessing memory function. Abnormal fiber structures that demonstrated significant correlations with serum GDNF levels included the left internal capsule, left corticospinal tract, right corpus callosum, and right cingulate gyrus.

**Conclusion:**

The decrease in serum GDNF levels among PD-MCI patients exhibiting impairments in attention and working memory function was significantly correlated with alterations in the corpus callosum (knee) and posterior cingulate gyrus. Furthermore, the reduction of serum GDNF levels in PD-MCI patients with impaired executive function is associated with changes in the internal capsule (forelimb) projection fibers. Additionally, the decline of serum GDNF levels in PD-MCI patients experiencing memory function impairment is related to alterations in the right cingulate gyrus.

## 1 Introduction

PD-MCI is a common non-motor symptom observed in the early stages of Parkinson’s disease, with over 50% of PD-MCI cases progressing to Parkinson’s disease dementia (PDD) (Counsell et al., 2022). This progression significantly affects the prognosis and quality of life for those impacted. Currently, there are no definitive biomarkers available for the early diagnosis and intervention in disease progression related to PD-MCI (Miller and O’Callaghan, 2015). Therefore, investigating the underlying mechanisms of PD-MCI is crucial for facilitating early detection and treatment of Parkinson’s disease.

Studies have indicated that the brain damage observed in patients with PD is primarily “subcortical,” suggesting that abnormalities in the structure of white matter fibers may play a significant role in the onset and progression of cognitive dysfunction associated with PD (Hou and Shang, 2022). Emerging evidence has suggested that alterations in white matter during the early stages of PD not only facilitate the detection of various motor symptoms but also enhance our understanding of the neural underpinnings related to clinical markers indicative of precursors to PD, such as rapid eye movement (REM) sleep behavior disorder and olfactory dysfunction (Ansari et al., 2016; Rahmani and Aarabi, 2017). Furthermore, studies have demonstrated that white matter abnormalities in PD patients are directly correlated with a reduction in dopaminergic transporters when compared to normally aging adults (Zhang et al., 2016).

GDNF exerts a significant neurotrophic effect on dopaminergic neurons. It promotes the survival, morphological differentiation, damage repair, and dopamine release of these neurons while also regulating their excitability in the midbrain (Wang et al., 2011; Brown et al., 2018). Research has demonstrated that intraventricular injection of GDNF can enhance spatial learning and memory functions in rats (Pertusa et al., 2008). Furthermore, studies indicate that the concentration of GDNF in the brain decreases concomitantly with declines in learning and memory functions in rats suffering from nerve infections (Gui et al., 2017). Jaan-Olle Andressoo team discovered that a sustained two-fold increase in endogenous GDNF safely elevated dopamine levels and enhanced motor learning capabilities in both young and aged mice (Mätlik et al., 2018; Turconi et al., 2020). Our previous investigations revealed that serum levels of GDNF were significantly reduced in patients with PD-MCI compared to a normal control group. This reduction may contribute to impairments in attention, memory, and executive function among PD-MCI patients, potentially acting independently or synergistically with neurotransmitters (Liu et al., 2022; Tong et al., 2023).

Since alterations in white matter occur early in PD and may reflect the progression of symptoms (Haghshomar et al., 2019), coupled with the significant decrease in serum GDNF levels observed in PD-MCI patients, we posed the question of whether serum GDNF could predict changes in white matter fibers among individuals with PD-MCI. In this study, we quantitatively examined the modifications in brain white matter fiber structure within PD-MCI patients and performed a correlation analysis with serum GDNF levels. This approach aims to further investigate the role of GDNF in the onset and progression of PD-MCI while seeking potential biomarkers for screening this condition. Ultimately, our findings may provide objective foundations for clinical screening and diagnosis of PD-MCI at its early stages.

## 2 Materials and methods

### 2.1 Subjects

We recruited a total of 105 PD outpatients and inpatients from the Neurology Department of the Affiliated Hospital of Xuzhou Medical University, spanning the period from January 2018 to December 2020.

Inclusion criteria: (1) participants were required to be between the ages of 40 and 80 years old; (2) participants needed to demonstrate the ability to successfully complete all cognitive tests as directed by a medical professional, and should not have experienced any difficulties in listening, understanding, or writing; (3) the diagnosis of PD was independently confirmed by two experienced neurologists using the UK PD Society Brain Bank Clinical Diagnostic Criteria (Hughes et al., 1993); (4) written informed consent was obtained from either the patients themselves or their legal representatives.

Exclusion criteria: (1) with neurological histories other than PD, e.g., moderate or serious brain injury, stroke, and vascular dementia confirmed by CT/MRI; (2) secondary parkinsonism induced by drugs, vascular lesions, tumors, trauma, and other insults, Parkinsonism plus syndrome such as the progressive supranuclear palsy and multiple system atrophy; (3) major psychological diseases such as anxiety, depression and schizophrenia (Stuart et al., 2020); (4) systemic disease affecting the heart, liver or kidney, and other diseases that might affect cognitive function (Gonzalez-Redondo et al., 2014).

In addition, we enlisted a group of healthy individuals who were aging normally, ensuring that their age, sex, and education level were comparable to those of the PD patients as the healthy control (HC) group. Detailed materials and methods were described in our previous research (Liu et al., 2022).

This study was approved by the ethics committee of the Affiliated Hospital of Xu-zhou Medical University (approval no: XYFY2017-KL047-01). Written informed consents were signed by all subjects for enrollment.

### 2.2 Clinical evaluations

Clinical and demographic information, encompassing education, disease duration, medication, and family history, was gathered from all participants. Depression was evaluated using the Depression Rating Scale (GDS-30), while the severity of motor symptoms was assessed through the revised Unified Parkinson’s Disease Rating Scale (UPDRS III) (Goetz et al., 2010). Additionally, the stage of the disease was determined using the modified Hoehn and Yahr (H&Y) (Hoehn and Yahr, 1998).

### 2.3 Neuropsychological assessment

The global cognitive function was assessed with the Mini Mental State Examination (MMSE) and the Montreal Cognitive Assessment (MoCA) (Nasreddine et al., 2005). Five cognitive domains (attention and working memory, executive function, language, memory, and visuospatial functions) were determined using neuropsychological tests. Attention and working memory were assessed with the DSB-T (Wechsler, 1981) and TMT-A (Corrigan and Hinkeldey, 1987; Arbuthnott and Frank, 2000). Executive function was assessed with TMT-B (Corrigan and Hinkeldey, 1987; Arbuthnott and Frank, 2000) and CDT (Royall et al., 1998; Powlishta et al., 2002). Language was tested by the Boston Naming Test (BNT) (Kaplan et al., 1983) and Verbal Fluency Test (VFT) (Shao et al., 2014). The evaluation of memory included the 3-word recall test of MMSE (Dubois et al., 2017) and the AVLT-H version (Lavoie et al., 2018). Spatial function was assessed using the pentagon copying subset of the MMSE (using a modified 0 to 2 rating scale) (Ala et al., 2001) and the Clock Copying Test (CCT) (Royall et al., 1998; Powlishta et al., 2002).

All patients were evaluated in the “ON” state, and those with scores 1.5 standard deviations lower than the HC group were considered to have neuropsychological impairment. PD patients were classified using the diagnostic criteria recommended by the Movement Disorder Society (MDS) with literature support as PD-MCI (*n* = 52) and PD-N (*n* = 53).

### 2.4 Blood sampling

Blood samples from overnight fasting subjects were collected between 8:00 am and 9:00 am, followed by centrifugation at 2,000 × *g* for 15 min, aliquoted, and stored at −80°C before use.

### 2.5 Determination of serum GDNF levels

Serum GDNF levels were measured with enzyme-linked immunosorbent assay (ELISA) (R&D Systems, USA).

### 2.6 DTI data acquisition

MRI scanning was performed on Signa Excite GE 3.0T system with a 8-channel SENSE head coil. For DTI, diffusion-weighted echo-planar imaging (DW-EPI) was used with the following parameters: repetition time (TR) = 10,000 ms, echo time (TE) = 76 ms, field of view (FOV) = 320 mm^2^, matrix size 128 × 128, NEX = 2, slice spacing = 2.5 mm with no gap. The b values was 0 and 1,000 s/mm^2^, respectively, and the diffusion sensitive gradient direction was 25. The eFilm Workstation was used to transfer and save the original DTI image file.

### 2.7 DTI data analysis

DTI was employed to perform whole-brain scanning. The entire brain image was segmented into small voxel (volume pixel) units using the Voxel-Based Analysis (VBA) method. A quantitative and automated analysis of the whole brain was conducted based on voxel density, so as to achieve the objective, sensitive, and quantitative assessment of changes in the internal microstructure of the brain. The DTI data were divided into two segments. In the first segment, the VBA method was employed to compare whole brain white matter FA values and identify brain regions exhibiting significant differences in FA between the two groups. In the second segment, the FA values of specific brain areas were extracted based on the regions identified in the first segment.

The data preprocessing was performed using PANDA^1^ (Cui et al., 2013). Converted the original DICOM image to 4D NIFTI format image file and obtained the gradient encoding files .bval and .bvec for DTI scanning. Skull stripping with the brain extraction tool (BET) was applied in each subject. Eddy current correction was used to correct for distortions and head motion on the DTI sequences by aligning the diffusion weighted images to the *b* = 0 image. Constructed a diffusion tensor model using the dtifit function of FSL, and calculated the voxel level FA value based on the tensor eigenvalues lambda of the three main directions. Normalize the FA graph space in a linear and nonlinear manner. Select the registered FA image for Gaussian smoothing, using a FWHM (full width at half maximum) value of 6 mm to improve the signal-to-noise ratio of the image. Selected the second-order analysis in SPM8, established the spm files of ANOVA and independent sample *t*-test, respectively, and estimated the model, defined the design matrix and reported the results. Statistical results of SPM8 were displayed and anatomically localized on a standard TIWI/T2W2 template using xjView 9.5.^2^ The region with significant changes in FA values obtained in the previous step was defined as ROIs, and individual ROI was directly defined on xjView, and the restplus toolkit was used to extract FA values.

### 2.8 Statistical analysis

SPSS 16.0 software was used for statistical analysis. A normality test was performed, and normally distributed data were presented as mean ± standard deviation (X ± SD), while non-normal data were expressed as median (interquartile range). *T*-tests were used to compare between two groups. For multigroup comparison, parametric data were analyzed using analysis of variance (ANOVA), and non-parametric data were compared using the Kruskal-Wallis test, with the LSD method (or Bonferroni method) used for multiple comparisons between groups. Correlation analysis was carried out using either the Pearson or Spearman correlation coefficient, depending on the distribution of variables. A two-sided *P* < 0.05 was considered statistical significance.

## 3 Results

### 3.1 Analysis of demographic data and clinical characteristics of each group

A total of PD patients were included in the study and classified into two groups: PD patients with normal cognitive function (PD-N, *n* = 52), PD patients with MCI (PD-MCI, *n* = 53), and were compared with healthy control (HC, *n* = 45) for general demographic data as well as MMSE, MoCA and GDS-30 scores (Table 1). There were no significant differences in age, sex ratio, and education level among the three groups (*P* > 0.05). PD patients had lower MMSE scores and more severe depressive symptoms compared to the HC group (MMSE: *H* = 78.216, *P* < 0.001; MoCA: *H* = 80.167, *P* < 0.001; GDS-30, F_2,150_ = 9.258, *P* < 0.001). In addition, we compared the clinical manifestations (UPDRS-III and Hoehn-Yahr grades) and disease duration in the PD-N and PD-MCI groups (Table 1), and observed a decline in cognitive ability as the UPDRS-III score (*P* < 0.001) and Hoehn-Yahr grade (*P* < 0.001) increased. The serum GDNF level showed significant variation among the three groups (*P* < 0.001), with the PD-MCI group exhibiting significantly lower levels compared to the PD-N group and HC group (*P* < 0.05).

**TABLE 1:** Demographics and clinical characteristics of each group.

	HC (*n* = 45)	PD-N (*n* = 52)	PD-MCI (*n* = 53)	*P*-value
Age (years)	63.40 ± 8.17	64.45 ± 8.15	64.02 ± 9.70	0.051
Education (years)	9.18 ± 2.14	10.17 ± 2.49	10.09 ± 2.14	0.082
Sex, M/F	4/6	5/5	4/6	0.680
MMSE score	29 (28–29)	28.5 (27–29)	26 (26–27)	< 0.001
MoCA score	26 (25–28)	25 (24–27)	23 (22–24)	< 0.001
GDS-30 score	6.28 ± 3.91	9.16 ± 5.21	7.19 ± 3.19	< 0.001
UPDRS-III score	NA	19.32 ± 7.93	25.37 ± 9.00	< 0.001
Hoehn-Yahr grade	NA	1.75 (1–2)	2.5 (2–2.5)	< 0.001
Disease duration (months)	NA	24 (11–60)	24 (12–72)	0.024
GDNF (pg/ml)	573.51 ± 89.50	455.56 ± 79.24	384.44 ± 76.24	< 0.001

PD-N, PD with normal cognitive function; PD-MCI, PD with mild cognitive impairment; HC, normal control; MMSE, mini-mental state examination; MoCA, Montreal Cognitive Assessment; GDS-30, Geriatric Depression Scale-30; UPDRS-III, unified PD rating scale part III exercise evaluation; H-Y, Hoehn-Yahr grade; GDNF, glial cell line-derived neurotrophic factor. Data were represented as X ± SD or median (interquartile range).

### 3.2 Comparison of neuropsychological assessments in each group

In all neuropsychological assessments performed, statistically significant differences were observed between groups (*P* < 0.001) (Table 2). Further analysis comparing the two groups of PD patients revealed that the neuropsychological assessment results of the PD-MCI group differed significantly from those of the PD-N group, except for the CCT (PD-N vs. PD-MCI, *P* = 0.250). However, no significant differences in neuropsychological assessment results were found between the PD-N group and the HC group (*P* > 0.05).

**TABLE 2:** Neuropsychological assessment results for healthy controls and Parkinson’s disease patients.

	HC (*n* = 45)	PD-N (*n* = 52)	PD-MCI (*n* = 53)	*P*-value
DSB-T (n)	4 (4–5)	4 (3–5)	3 (3–4)	< 0.001
TMT-A(s)	57.41 ± 24.15	61.32 ± 19.51	86.20 ± 20.52	< 0.001
TMT-B(s)	128.14 ± 35.15	115.70 ± 30.57	200.58 ± 29.69	< 0.001
CDT	4 (4–4)	4 (4–4)	3 (2–4)	< 0.001
BNT (n)	24.13 ± 3.12	22.18 ± 2.96	18.92 ± 3.19	< 0.001
SFT (n)	15.03 ± 2.48	15.86 ± 3.06	12.28 ± 2.29	< 0.001
AVLT-H (n)	16.97 ± 4.28	17.16 ± 3.97	12.04 ± 4.12	< 0.001
3-Word recall of the MMSE	3 (3–3)	3 (2.5–3)	2 (1–3)	< 0.001
Intersecting pentagons from the MMSE (0–2)	2 (2–2)	2 (2–2)	1 (0–1)	< 0.001
CCT	4 (4–4)	4 (4–4)	4 (3–4)	< 0.001

PD-N, PD with normal cognitive function; PD-MCI, PD with mild cognitive impairment; HC, normal control; MMSE, mini-mental state examination; Data were represented as X ± SD or median (interquartile range).

### 3.3 Comparison of FA values of white matter fibers in different groups

**FIGURE 1:**
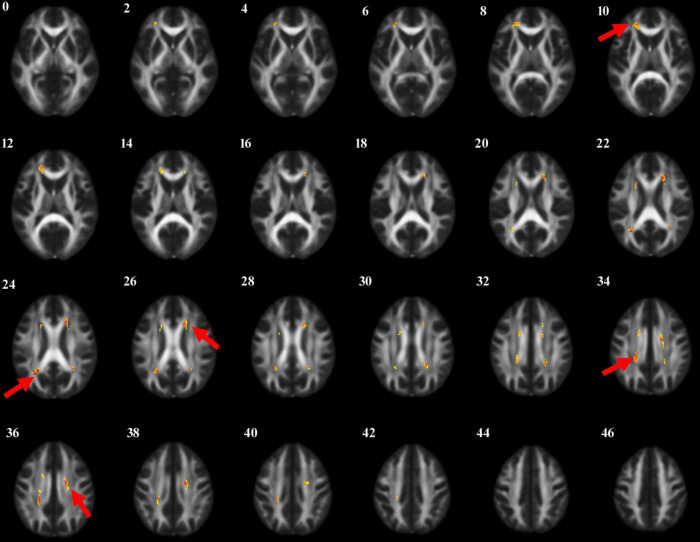
The anatomic locations of the regions with decreased FA value in HC, PD-N and PD-MCI groups were compared (*P* < 0.001). The arrows indicate the location of the brain regions with decreased FA values on the high-resolution T1WI map, shown as white/yellow/red markers with increasing intensity.

**FIGURE 2:**
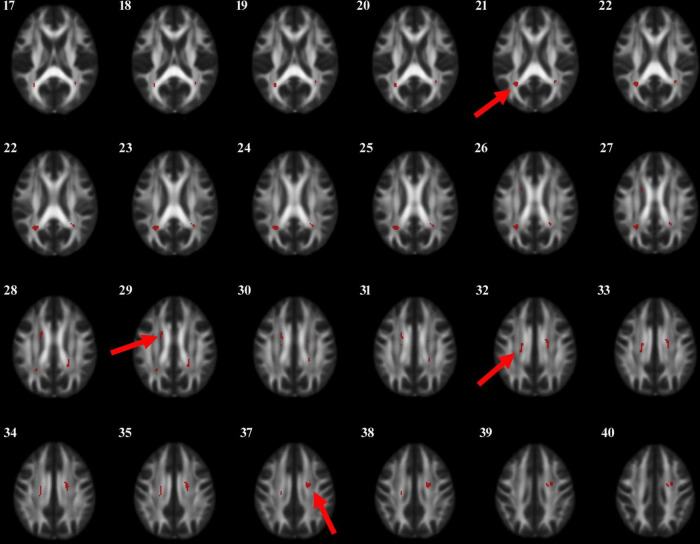
The anatomic locations of the regions with decreased FA value were compared between PD-N group and PD-MCI group (*P* < 0.001). The arrows indicate the location of the brain regions with decreased FA values on the high-resolution T1WI map, shown as white/yellow/red markers with increasing intensity.

Through voxel-based comparison of the whole brain (*P* < 0.001), FA values were reduced in the left corticospinal tract, the right internal capsule, the left corpus callosum, and the right cingulate gyrus in all three groups (Table 3 and Figure 1). The areas where FA values decreased in the PD-MCI group compared to the PD-N group were the left corpus callosum, right corpus callosum, left corticospinal tract, right cingulate gyrus, and left internal capsule (Table 4 and Figure 2).

**TABLE 3:** Comparison of anatomical locations in areas with decreased FA values among three groups.

Coordinate			Anatomical position	*P*-value	Cluster-value (mm^3^)
**X**	**Y**	**Z**			
−28	−56	22	Corticospinal tract-left	< 0.001	464
30	−52	22	Internal capsule-right	< 0.001	240
−20	2	28	Corpus callosum-left	< 0.001	136
−20	−22	34	Corpus callosum-left	< 0.001	184
18	−10	34	Cingulate gyrus-right	< 0.001	376

The FA value decreased in HC, PD-N and PD-MCI groups (*P* < 0.001); Cluster size, representing the voxel value of the difference region, in cubic millimeters.

**TABLE 4:** Anatomic location of the decreased FA value between PD-N group and PD-MCI group.

Coordinate			Anatomical position	*P*-value	Cluster-value (mm^3^)
**X**	**Y**	**Z**			
−18	40	10	Corpus callosum-left	< 0.001	632
16	20	24	Corpus callosum-right	< 0.001	680
−28	−58	24	Corticospinal tract-left	< 0.001	400
−20	12	24	Corpus callosum-left	< 0.001	136
22	−44	30	Cingulate gyrus-right	< 0.001	336
−18	2	30	Corpus callosum-left	< 0.001	208
−22	−32	36	Internal capsule-left	< 0.001	544
18	−8	36	Corpus callosum-right	< 0.001	520

Compared with the PD-N group, the brain regions with decreased FA values in the PD-MCI group (*P* < 0.001); Cluster size, representing the voxel value of the difference region, in cubic millimeters.

### 3.4 Correlation analysis between neuropsychological assessments and abnormal white matter fiber FA values in PD-MCI patients

In order to screen for brain regions that are significantly associated with cognitive impairment in PD-MCI patients, we analyzed the correlation between neuropsychological assessments and abnormal white matter fiber FA values in PD-MCI patients (Table 5). The results showed that in PD-MCI patients, the regions significantly correlated with the DSB-T scores were the left and right corpus callosum (*P* = 0.031, *P* = 0.037) and the right cingulate gyrus (*P* = 0.007). The regions that exhibited a significant correlation with the TMT-A scores were the right corpus callosum (*P* = 0.029) and the right cingulate gyrus (*P* = 0.020). Additionally, the left and right internal capsule demonstrated a significant correlation with the TMT-B score (*P* = 0.018, *P* = 0.005). Furthermore, the left internal capsule displayed a significant correlation with the scores of the CDT (*P* = 0.04), while the right cingulate gyrus exhibited a significant association with the scores of the AVLT-H (*P* = 0.023).

**TABLE 5:** Anatomic location of abnormal brain areas associated with neuropsychological assessment scores in PD-MCI patients.

	Coordinate			Anatomical position	*r*-value
	**X**	**Y**	**Z**		
DSB-T (n)^a^	−18	40	10	Corpus callosum-left	0.459*
	18	−8	36	Corpus callosum-right	0.347*
	22	−44	30	Cingulate gyrus-right	0.714**
TMT-A(s)^b^	16	20	24	Corpus callosum-right	−0.392*
	18	−10	34	Cingulate gyrus-right	−0.388*
TMT-B(s)^b^	−22	−32	36	Internal capsule-left	−0.313*
	30	−52	22	Internal capsule-right	−0.676**
CDT^a^ AVLT-H (n)^b^	−22	−32	36	Internal capsule-left	0.289*
	22	−44	30	Cingulate gyrus-right	0.416*

*^a^*Spearman correlation coefficient;

*^b^*Pearson’s correlation coefficient.

*P < 0.05 and **P < 0.01.

### 3.5 Correlation analysis between serum GDNF levels and FA values of abnormal white matter fibers in PD-MCI patients

**FIGURE 3:**
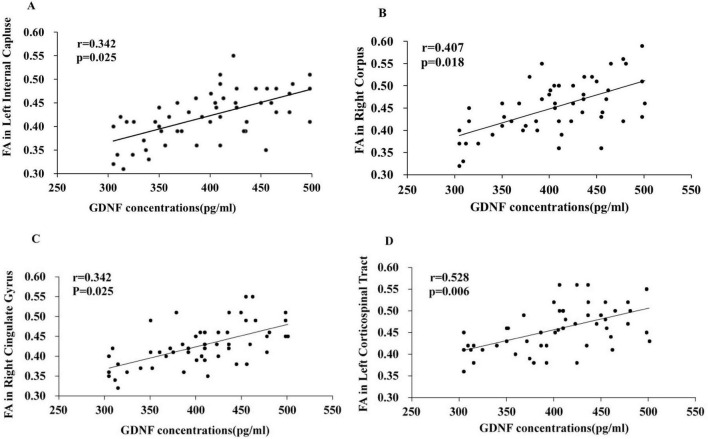
Correlation analysis between serum GDNF levels and abnormal brain white matter fibers FA values in PD-MCI patients. Correlation between serum GDNF concentration and the FA values of left inner capsule **(A)**, right corpus callosum **(B)**, right cingulate gyrus **(C)**, left corticospinal tract **(D)**.

The correlation analysis between serum GDNF levels and abnormal white matter fiber FA values was performed on the PD-MCI group to screen brain regions significantly correlated with changes in serum GDNF levels in PD-MCI patients, and the results showed (Figure 3): in the PD-MCI group, the regions that showed a significant correlation with GDNF levels were the left internal capsule (*r* = 0.342, *P* = 0.025), right corpus callosum (*r* = 0.407, *P* = 0.018), right cingulate gyrus (*r* = 0.655, *P* = 0.001), and left corticospinal tract (*r* = 0.528, *P* = 0.006). However, there was no significant correlation with other abnormal white matter fibers (*P* > 0.05). Additionally, the serum levels of GDNF in the HC and PD-N groups did not show a significant correlation with abnormal white matter fibers (*P* > 0.05).

## 4 Discussion

Mild cognitive impairment (MCI) is observed in the early stages of PD, prior to the onset of motor symptoms. This study, utilizing neuropsychological assessments, investigates for the first time the correlation between serum GDNF levels and alterations in brain white matter among PD-MCI patients. The findings indicate that decreased serum GDNF levels in PD-MCI patients with executive function impairments are significantly correlated with changes in the internal capsule projection fibers. Furthermore, reduced serum levels of GDNF in PD-MCI patients who exhibit deficits in attention and working memory are significantly correlated with alterations in both the corpus callosum and cingulate gyrus. We propose that serum GDNF levels may serve as a potential biological marker for the early prediction of brain white matter changes in PD-MCI patients, providing a convenient, non-invasive, and cost-effective tool for the early screening of Parkinson’s disease.

Considering the impact of factors such as medication use and disease staging on research indicators, we strictly followed the inclusion and exclusion criteria in the collection of research subjects Furthermore, we conducted a comparative analysis of population data and clinical characteristics among patients to avoid the influence of differences in basic characteristics of patients within the group.

GDNF functions as a neuron-specific neuroprotective factor for dopamine neurons, it promotes their survival, morphological differentiation, and repair following damage. Previous research has demonstrated that concentrations of GDNF in cerebrospinal fluid from PD patients are lower than those found in normal controls (Vaillancourt et al., 2013). Consistent with earlier clinical studies (Garbayo et al., 2016), our study also revealed that peripheral serum GDNF levels were diminished in PD patients compared to healthy controls. Numerous experiments have identified GDNF as a promising therapeutic candidate for addressing motor symptoms associated with PD. The research team proposed that the survival of adult nigrostriatal dopaminergic neurons is critically dependent on the production of GDNF in the striatum. Selective pharmacological modulation of GDMF by striatal parvalbumin interneurons is a promising therapeutic strategy to stimulate endogenous production of GDNF in the striatum combat neuronal death in Parkinson’s disease (Hidalgo-Figueroa et al., 2012; Kumar et al., 2015; Enterría-Morales et al., 2020). However, its application within the cognitive domain-particularly concerning learning and memory functions–remains largely confined to animal studies. Our previous investigations have established significant correlations between serum GDNF levels and scores on tasks such as DSB-T, TMT-A, and TMT-B among PD-MCI patients (Liu et al., 2022).

DTI is an advanced technique that enhances and expands upon diffusion-weighted imaging methodologies (Hattori et al., 2012; Taylor et al., 2018). In the current investigation, we examined alterations in FA and mean diffusivity (MD) across various cerebral white matter tracts using DTI. However, no statistically significant differences were observed in the MD values; therefore, these data have been excluded from the results.

Research has indicated a more widespread correlation between attention and working memory capacity with microstructural changes in brain white matter (Kamagata et al., 2012; Brown et al., 2023; Meng et al., 2022). The cingulate gyrus connects to the hippocampus, parahippocampal gyrus, and anterior cingulate gyrus via the cingulate tracts, playing a crucial role in regulating learning, memory, and emotion (Yang et al., 2023; Wu and Zhang, 2023). A decrease in FA values within the cingulate gyrus suggests abnormal fiber connectivity of this structure. As the largest white matter pathway in the brain, alterations within the corpus callosum are evident both during normal aging and senile cognitive decline. Changes in diffusion metrics of the corpus callosum are associated not only with disruptions of motor circuits during cognitive assessments (Gallagher et al., 2013), but also appear more pronounced among Parkinson’s disease patients exhibiting postural instability (Hall et al., 2016). In this study, scores on DSB-T, TMT-A, and AVLT tests among PD-MCI patients showed significant correlations with changes observed in both the cingulate gyrus and corpus callosum. This further elucidates the role of white matter alterations in early Parkinson’s disease pathogenesis. Additionally, this study is pioneering in suggesting that decreased serum levels of GDNF among PD-MCI patients experiencing impairments in attention and working memory function correlate significantly with changes occurring within both the corpus callosum and cingulate gyrus.

Primary Parkinson’s disease is frequently associated with patterns of executive dysfunction that resemble those observed in patients with frontal lobe injuries, and this relationship is closely linked to the degeneration of the dopaminergic system. Recent studies have consistently demonstrated that the thickness of the prefrontal cortex, particularly in the dorsolateral prefrontal region, is reduced in PD patients. This reduction correlates with impairments in executive function (Mak et al., 2014; Koshimori et al., 2015). In this study, a significant correlation was identified between scores on the TMT-B and CDT and alterations in the internal capsule among PD-MCI patients. Notably, it was proposed for the first time that serum levels of GDNFare significantly correlated with changes in the internal capsule among PD-MCI patients exhibiting impaired executive function. This phenomenon is thought to arise from lesions within the internal capsule, which disrupts connectivity between the frontal cortex and thalamus, ultimately leading to compromised functionality of both regions-the frontal lobe and internal capsule (Ban et al., 2019).

This study proposed the early predictive value of serum GDNF levels for white matter changes in patients with PD-MCI and speculated on its potential mechanisms of involvement. One possibility is that GDNF may synergistically contribute to cognitive processes by regulating neuronal survival and synaptic plasticity. Previous research has demonstrated that GDNF promotes the differentiation of exogenous neural stem cells into preoligodendrocytes, thereby facilitating white matter myelination (Duarte et al., 2012). Additionally, it plays a crucial role in endogenous white matter self-repair following ischemia by enhancing the survival rate of white matter and subventricular zone neuroglial cells (Li et al., 2015). Another potential mechanism is that GDNF may counteract neuronal degeneration and delay the onset of cognitive decline. The specific pathways involved remain incompletely understood but may relate to GDNF’s ability to mitigate oxidative stress, inhibit microglial activation, and reduce neuroinflammation. It is important to note that the mechanisms underlying GDNF’s role in the emergence and progression of early cognitive impairment in Parkinson’s disease are complex; thus, further comprehensive studies are warranted.

## 5 Conclusion

Under our experimental conditions, this study found that the decrease in serum GDNF levels among PD-MCI patients with impairments in attention and working memory function was significantly correlated with alterations in the corpus callosum and cingulate gyrus. Additionally, the reduction of serum GDNF levels in PD-MCI patients exhibiting impaired executive function is associated with changes in the internal capsule projection fibers. Owing to the constraints of funds and time, the research subjects were merely collected in the Affiliated Hospital of Xuzhou Medical University, which gives rise to certain limitations for this study. In future research, we intend to enlarge the sample size and carry out longitudinal studies to further clarify the underlying mechanism by combining a comprehensive approach encompassing animal models, cell experiments and functional imaging techniques. Despite its limitations, this study proposes the potential role of GDNF as an early diagnostic marker for Parkinson’s disease, aiming to provide a more robust theoretical foundation for subsequent clinical practice.

## Data Availability

The original contributions presented in this study are included in the article/supplementary material, further inquiries can be directed to the corresponding author.

## Footnotes

1
http://www.nitrc.org/projects/panda/

2
http://www.alivelearn.net/xjview/

## References

[B1] Ala T. A. Hughes L. F. Kyrouac G. A. Ghobrial M. W. Elble R. J. (2001). Pentagon copying is more impaired in dementia with Lewy bodies than in Alzheimer’s disease. *J. Neurol. Neurosurg. Psychiatry* 70 483–488. 10.1136/jnnp.70.4.483 11254771 PMC1737321

[B2] Ansari M. Rahmani F. Dolatshahi M. Pooyan A. Aarabi M. H. (2016). Brain pathway differences between Parkinson’s disease patients with and without REM sleep behavior disorder. *Sleep Breath* 21 155–161. 10.1007/s11325-016-1435-8 27853964

[B3] Arbuthnott K. Frank J. (2000). Trail making test, part B as a measure of executive control: Validation using a set-switching paradigm. *J. Clin. Exp. Neuropsychol.* 22 518–528. 10.1076/1380-3395 10923061

[B4] Ban S. Wang H. Wang M. Xu S. Qin Z. Su J. (2019). Diffuse tract damage in CADASIL is correlated with global cognitive impairment. *Eur. Neurol.* 81 294–301. 10.1159/000501612 31484188

[B5] Brown G. Hakun J. Lewis M. M. De J. S. Du G. Eslinger P. J. (2023). Frontostriatal and limbic contributions to cognitive decline in Parkinson’s disease. *J. Neuroimaging* 33 121–133. 36068704 10.1111/jon.13045 PMC9840678

[B6] Brown R. W. Schlitt M. A. Owens A. S. DePreter C. C. Cummins E. D. Kirby S. L. (2018). Effects of environmental enrichment on nicotine sensitization in rats neonatally treated with quinpirole: Analyses of glial cell line-derived neurotrophic factor and implications towards schizophrenia. *Dev. Neurosci.* 40 64–72. 10.1159/000486391 29444518

[B7] Corrigan J. D. Hinkeldey N. S. (1987). Relationships between parts A and B of the trail making test. *J. Clin. Psychol.* 43 402–414. 3611374 10.1002/1097-4679(198707)43:4<402::aid-jclp2270430411>3.0.co;2-e

[B8] Counsell C. Giuntoli C. Khan I. Maple-Grødem J. Macleod A. D. (2022). The incidence, baseline predictors, and outcomes of dementia in an incident cohort of Parkinson’s disease and controls. *J. Neurol.* 269 4288–4298. 10.1007/s00415-022-11058-2 35307754 PMC9294013

[B9] Cui Z. Zhong S. Xu P. He Y. Gong G. (2013). PANDA: A pipeline toolbox for analyzing brain diffusion images. *Front. Hum. Neurosci.* 7:42. 10.3389/fnhum.2013.00042 23439846 PMC3578208

[B10] Duarte E. P. Curcio M. Canzoniero L. M. Duarte C. B. (2012). Neuroprotection by GDNF in the ischemic brain. *Growth Factors* 30 242–257. 10.3109/08977194.2012.691478 22670840

[B11] Dubois B. Burn D. Goetz C. Aarsland D. Brown R. G. Broe G. A. (2017). Diagnostic procedures for Parkinson’s disease dementia: Recommendations from the movement disorder society task force. *Mov. Disord.* 22 2314–2324. 10.1002/mds.21844 18098298

[B12] Enterría-Morales D. Del Rey N. Blesa J. López-López I. Gallet S. Prévot V. (2020). Molecular targets for endogenous glial cell line-derived neurotrophic factor modulation in striatal parvalbumin interneurons. *Brain Commun.* 27:fcaa105. 10.1093/braincomms/fcaa105 32954345 PMC7472905

[B13] Gallagher C. Bell B. Bendlin B. Palotti M. Okonkwo O. Sodhi A. (2013). White matter microstructural integrity and executive function in Parkinson’s disease. *J. Int. Neuropsychol. Soc.* 19 349–354. 10.1017/S1355617712001373 23321049 PMC3637933

[B14] Garbayo E. Ansorena E. Lana H. Carmona-Abellan M. D. Marcilla I. Lanciego J. L. (2016). Brain delivery of microencapsulated GDNF induces functional and structural recovery in parkinsonian monkeys. *Biomaterials* 110 11–23. 10.1016/j.biomaterials.2016.09.015 27697668

[B15] Goetz C. G. Fahn S. Martinez-Martin P. Poewe W. Sampai C. (2010). Movement disorder society -sponsored revision of the Unified Parkinson’s disease rating scale (MDS-UPDRS): Process, format, and clinimetric testing plan. *Mov. Disord.* 23 2129–2170. 10.1002/mds.21198 17115387

[B16] Gonzalez-Redondo R. García-García D. Clavero P. Gasca-Salas C. GarcíaEulate R. Zubieta J. L. (2014). Grey matter hypometabolism and atrophy in Parkinson’s disease with cognitive impairment: A two-step process. *Brain* 137 2356–2367. 10.1093/brain/awu159 24951642 PMC4610189

[B17] Gui L. Lei X. Zuo Z. (2017). Decrease of glial cell-derived neurotrophic factor contributes to anesthesia- and surgery-induced learning and memory dysfunction in neonatal rats. *J. Mol. Med.* 95 369–379. 10.1007/s00109-017-1521-9 28213636

[B18] Haghshomar M. Rahmani F. Hadi Aarabi M. Shahjouei S. Sobhani S. Rahmani M. (2019). White matter changes correlates of peripheral neuroinflammation in patients with Parkinson’s disease. *Neuroscience* 403 70–78. 10.1016/j.neuroscience.2017.10.050 29126955

[B19] Hall J. M. Ehgoetz Martens K. A. Walton C. C. O’Callaghan C. Keller P. E. Lewis S. J. (2016). Diffusion alterations associated with Parkinson’s disease symptomatology: A review of the literature. *Parkinsonism Relat. Disord.* 33 12–26. 10.1016/j.parkreldis.2016.09.026 27765426

[B20] Hattori T. Orimo S. Aoki S. Ito K. Abe O. Amano A. (2012). Cognitive status correlates with white matter alteration in Parkinson’s disease. *Hum. Brain Mapp.* 33 727–739. 21495116 10.1002/hbm.21245 PMC6870034

[B21] Hidalgo-Figueroa M. Bonilla S. Gutiérrez F. Pascual A. López-Barneo J. (2012). GDNF is predominantly expressed in the PV+ neostriatal interneuronal ensemble in normal mouse and after injury of the nigrostriatal pathway. *J. Neurosci.* 18 864–872. 10.1523/JNEUROSCI.2693-11.2012 22262884 PMC6621168

[B22] Hoehn M. M. Yahr M. D. (1998). Parkinsonism: Onset, progression and mortality. *Neurology* 50 11–26. 10.1212/wnl.50.2.318 9484345

[B23] Hou Y. Shang H. (2022). Magnetic resonance imaging markers for cognitive impairment in Parkinson’s disease: Current view. *Front. Aging Neurosci.* 14:788846. 10.3389/fnagi.2022.788846 35145396 PMC8821910

[B24] Hughes A. J. Daniel S. E. Blankson S. Lees A. J. (1993). A clinicopathologic study of 100 cases of Parkinson’s disease. *Arch. Neurol.* 50 140–148. 10.1001/archneur.1993.00540020018011 8431132

[B25] Kamagata K. Motoi Y. Abe O. (2012). White matter alteration of the cingulum in parkinson disease with and without dementia: Evaluation by diffusion tensor tract-specific analysis. *Am. J. Neuroradiol.* 33 890–895. 22241380 10.3174/ajnr.A2860 PMC7968830

[B26] Kaplan E. F. Goodglass H. Weintraub S. (1983). *The Boston Naming Test*, 2nd Edn. Philadelphia: Lea and Febiger.

[B27] Koshimori Y. Segura B. Christopher L. Lobaugh N. Duff-Canning S. Mizrahi R. (2015). Imaging changes associated with cognitive abnormalities in Parkinson’s disease. *Brain Struct. Funct.* 220 2249–2261. 10.1007/s00429-014-0785-x 24816399 PMC4485490

[B28] Kumar A. Kopra J. Varendi K. Porokuokka L. Panhelainen A. Kuure S. (2015). GDNF overexpression from the native locus reveals its role in the nigrostriatal dopaminergic system function. *PLoS Genet.* 17:e1005710. 10.1371/journal.pgen.1005710 26681446 PMC4682981

[B29] Lavoie M. Bherer L. Joubert S. Gagnon J. F. Blanchet S. Rouleau I. (2018). Normative data for the Rey auditory verbal learning test in the older french-quebec population. *Clin. Neuropsychol.* 32 15–28. 10.1080/13854046.2018.1429670 29388473

[B30] Li W. J. Mao F. X. Chen H. J. Qian L. H. Buzby J. S. (2015). Treatment with UDP-glucose, GDNF, and memantine promotes SVZ and white matter self-repair by endogenous glial progenitor cells in neonatal rats with ischemic PVL. *Neuroscience* 284 444–458. 10.1016/j.neuroscience.2014.10.012 25453769

[B31] Liu Y. Tong S. Ding L. Liu N. Gao D. (2022). Serum levels of glial cell linederived neurotrophic factor and multiple neurotransmitters: In relation to cognitive performance in Parkinson’s disease with mild cognitive impairment. *Int. J. Geriatr. Psychiatry* 35 153–162. 10.1002/gps.5222 31650626

[B32] Mak E. Zhou J. Tan L. C. S. Au W. Sitoh Y. Kandiah N. (2014). Cognitive deficits in mild Parkinson’s disease are associated with distinct areas of grey matter atrophy. *J. Neurol. Neurosurg. Psychiatry* 85 576–580. 10.1136/jnnp-2013-305805 24133286

[B33] Mätlik K. Võikar V. Vilenius C. Kulesskaya N. Andressoo J. (2018). Two-fold elevation of endogenous GDNF levels in mice improves motor coordination without causing side-effects. *Sci. Rep.* 8:11861. 10.1038/s41598-018-29988-1 30089897 PMC6082872

[B34] Meng L. Wang H. Zou T. Wang X. Chen H. Xie F. (2022). Attenuated brain white matter functional network interactions in Parkinson’s disease. *Hum. Brain Mapp.* 43 4567–4579. 35674466 10.1002/hbm.25973 PMC9491278

[B35] Miller D. O’Callaghan J. P. (2015). Biomarkers of Parkinson’s disease: Present and future. *Metabolism* 64 S40–S46. 10.1016/j.metabol.2014.10.030 25510818 PMC4721253

[B36] Nasreddine Z. S. Phillips N. A. Bedirian V. Charbonneau S. Whitehead V. Collin I. (2005). The montreal cognitive assessment, MoCA: A brief screening tool for mild cognitive impairment. *J. Am. Geriatr. Soc.* 53 695–699. 10.1111/j.1532-5415 15817019

[B37] Pertusa M. García-Matas S. Mammeri H. Adell A. Rodrigo T. Mallet J. (2008). Expression of GDNF transgene in astrocytes improves cognitive deficits in aged rats. *Neurobiol. Aging* 29 1366–1379. 10.1016/j.neurobiolaging.2007.02.026 17399854

[B38] Powlishta K. K. Von Dras D. D. Stanford A. Carr D. B. Tsering C. Miller J. P. (2002). The clock drawing test is a poor screen for very mild dementia. *Neurology* 59 898–903. 10.1212/wnl.59.6.898 12297574

[B39] Rahmani F. Aarabi M. H. (2017). Does apolipoprotein A1 predict microstructural changes in subgenual cingulum in early Parkinson? *J. Neurol.* 264 684–693. 10.1007/s00415-017-8403-5 28168521

[B40] Royall D. R. Cordes J. A. Polk M. (1998). CLOX: An executive clock drawing task. *J. Neurol. Neurosurg. Psychiatry* 64 588–594. 9598672 10.1136/jnnp.64.5.588 PMC2170069

[B41] Shao Z. Janse E. Visser K. Meyer A. S. (2014). What do verbal fluency tasks measure? Predictors of verbal fluency performance in older adults. *Front. Psychol.* 5:772–784. 10.3389/fpsyg.2014.00772 25101034 PMC4106453

[B42] Stuart S. Morris R. Giritharan A. Quinn J. Nutt J. G. Mancini M. (2020). Prefrontal cortex activity and gait in parkinson’s disease with cholinergic and dopaminergic therapy. *Mov. Disord.* 35 2019–2027. 10.1002/mds.28214 32830901

[B43] Taylor K. I. Sambataro F. Boess F. Bertolino A. Dukart J. (2018). Progressive decline in gray and white matter integrity in de novo parkinson’s disease: An analysis of longitudinal Parkinson progression markers initiative diffusion tensor imaging data. *Front. Aging Neurosci.* 10:318. 10.3389/fnagi.2018.00318 30349475 PMC6186956

[B44] Tong S. Y. Wang R. W. Li Q. Liu Y. Yao X. Y. Geng D. Q. (2023). Serum glial cell line-derived neurotrophic factor (GDNF) a potential biomarker of executive function in Parkinson’s disease. *Front. Neurosci.* 17:1136499. 10.3389/fnins.2023.1136499 36908789 PMC9995904

[B45] Turconi G. Kopra J. Võikar V. Kulesskaya N. Vilenius C. Piepponen T. (2020). Chronic 2-fold elevation of endogenous GDNF levels is safe and enhances motor and dopaminergic function in aged mice. *Mol. Ther. Methods Clin. Dev.* 17 831–842. 10.1016/j.omtm.2020.04.003 32368564 PMC7191127

[B46] Vaillancourt D. E. Schonfeld D. Kwak Y. Bohnen N. I. Seidler R. (2013). Dopamine overdose hypothesis: Evidence and clinical implications. *Mov. Disord.* 28 1920–1929. 10.1002/mds.25687 24123087 PMC3859825

[B47] Wang X. Hou Z. Yuan Y. Hou G. Liu Y. Li H. (2011). Association study between plasma GDNF and cognitive function in late-onset depression. *J. Affect. Disord.* 132 418–421. 10.1016/j.jad.2011.03.043 21524799

[B48] Wechsler D. (1981). *Wechsler Adult Intelligence Scale-Revised.* San Antonio: Harcourt.

[B49] Wu A. Zhang J. (2023). Neuroinflammation, memory, and depression: New approaches to hippocampal neurogenesis. *J. Neuroinflammation* 20:283. 10.1186/s12974-023-02964-x 38012702 PMC10683283

[B50] Yang J. Liang L. Wei Y. Liu Y. Li X. Huang J. (2023). Altered cortical and subcortical morphometric features and asymmetries in the subjective cognitive decline and mild cognitive impairment. *Front. Neurol.* 14:1297028. 10.3389/fneur.2023.1297028 38107635 PMC10722314

[B51] Zhang Y. Wu I. W. Tosun D. Foster E. Schuff N. (2016). Progression of regional microstructural degeneration in Parkinson’s disease: A multicenter diffusion tensor imaging study. *PLoS One* 31:e0165540. 10.1371/journal.pone.0165540 27798653 PMC5087900

